# Predictive Value of Glycosylated Hemoglobin for Post-operative Acute Kidney Injury in Non-cardiac Surgery Patients

**DOI:** 10.3389/fmed.2022.886210

**Published:** 2022-07-11

**Authors:** Lan-Ping Wu, Ke Pang, Bo Li, Yuan Le, Yong-Zhong Tang

**Affiliations:** ^1^Department of Anesthesiology, The Third Xiangya Hospital, Central South University, Changsha, China; ^2^Surgery Center, The Third Xiangya Hospital, Central South University, Changsha, China

**Keywords:** glycosylated hemoglobin, acute kidney injury, noncardiac surgery, predictor, cohort study

## Abstract

**Objective:**

Recent studies have indicated that patients (both with and without diabetes) with elevated hemoglobin A1c (HbA1c) have a higher rate of acute kidney injury (AKI) following cardiac surgery. However, whether HbA1c could help to predict post-operative AKI in patients after non-cardiac surgery is less clear. This study aims to explore the predictive value of pre-operative HbA1c for post-operative AKI in non-cardiac surgery.

**Methods:**

We reviewed the medical records of patients (≥ 18 years old) who underwent non-cardiac surgery between 2011 and 2020. Patient-related variables, including demographic and laboratory and procedure-related information, were collected, and univariable and multivariable logistic regression analyses were performed to determine the association of HbA1c with AKI. The area under the receiver operating curve (AUC), net reclassification improvement index (NRI), and integrated discriminant improvement index (IDI) were used to evaluate the predictive ability of the model, and decision curve analysis was used to evaluate the clinical utility of the HbA1c-added predictive model.

**Results:**

A total of 3.3% of patients (94 of 2,785) developed AKI within 1 week after surgery. Pre-operative HbA1c was an independent predictor of AKI after adjustment for some clinical variables (OR comparing top to bottom quintiles 5.02, 95% CI, 1.90 to 13.24, *P* < 0.001 for trend; OR per percentage point increment in HbA1c 1.20, 95% CI, 1.07 to 1.33). Compared to the model with only clinical variables, the incorporation of HbA1c increased the model fit, modestly improved the discrimination (change in area under the curve from 0.7387 to 0.7543) and reclassification (continuous net reclassification improvement 0.2767, 95% CI, 0.0715 to 0.4818, improved integrated discrimination 0.0048, 95% CI, -5e-04 to 0.0101) of AKI and non-AKI cases, NRI for non-AKI improvement 0.3222, 95% CI, 0.2864 to 0.3580 and achieved a higher net benefit in decision curve analysis.

**Conclusion:**

Elevated pre-operative HbA1c was independently associated with post-operative AKI risk and provided predictive value in patients after non-cardiac surgery. HbA1c improved the predictive power of a logistic regression model based on traditional clinical risk factors for AKI. Further prospective studies are needed to demonstrate the results and clinical application.

## Introduction

Acute kidney injury (AKI) is common and is associated with many adverse perioperative outcomes. It is a rapid damage to kidney function, occurring in 5.0–7.5% of all hospitalized patients and in up to 20% of critically ill patients (1). Post-operative AKI is relevant to surgical procedures. There is growing evidence that even mild, transient (lasting 24 h) AKI is interrelated with poorer long-term outcomes compared to people without AKI (2, 3). Patients with post-operative AKI exhibit delayed post-operative recovery, increased hospital mortality, and high medical costs (4, 5). Pre-operative identification of high-risk patients would allow earlier intervention and optimal perioperative management, thereby improving patient outcomes. Therefore, early identification and prevention of AKI are vital.

Diabetes mellitus (DM) has an increasing global prevalence; according to the International Diabetes Federation, in 2015, there were 415 million diabetes mellitus patients globally (6). Many studies have shown that DM is a recognized risk factor for AKI in non-cardiac surgeries (7–9). Glycosylated hemoglobin A1c (HbA1c), an important stand-alone diagnostic biomarker of diabetes mellitus, can reflect mean ambient fasting and postprandial glycemia over a 2–3 month period (10, 11). HbA1c is used to evaluate diabetes control and chronic hyperglycemia in diabetic patients (12). In addition, HbA1c is an important marker of insulin resistance, acute hyperglycemia risk, cardiovascular risk and atherosclerosis independent of the patient’s diabetic state (13, 14). Evidence indicates that HbA1c has predictive value for adverse outcomes after cardiovascular surgery (15–17). Recent studies have indicated that patients (both with and without diabetes) with elevated HbA1c have a higher rate of AKI following cardiac surgery (18–21). However, whether HbA1c can help to predict post-operative AKI in patients after non-cardiac surgery is less clear.

We hypothesized that HbA1c was associated with post-operative AKI and improved AKI prediction efficiency based on traditional clinical risk factors. This study aims to verify this hypothesis and provide evidence for the use of HbA1c to assess AKI risk prior to non-cardiac surgery.

## Materials and Methods

Ethics approval for this study (I 22025) was provided by the Ethics Committee of The Third Xiangya Hospital, Central South University, Changsha, Hunan, China. Informed consent was waived due to the retrospective design of the study. This study was conducted in accordance with a predefined protocol and statistical plan, which has been preregistered in the Chinese Clinical Trial Registry (ChiCTR2200057053). The statistical plan was not announced before the data analysis. This research followed the Strengthening the Reporting of Observational Studies in Epidemiology (STROBE) guidelines and the transparent reporting of a multivariable prediction model for individual prognosis or diagnosis (TRIPOD) statement.

The data for this study came from the hospital’s perioperative database, which was established by the Third Xiangya Hospital of Central South University. It contained anonymous demographic, medical, surgical, and laboratory information for all patients undergoing surgery in the anesthesiology department. All adult (18 years and older) patients had serum creatinine and HbA1c measurements within 30 days before surgery and at least one serum creatinine measurement within 7 days after surgery. For patients with multiple operations, only the first operation during the study period was included. The exclusion criteria were patients undergoing cardiac surgery, transplant surgery, urological surgery, previous myocardial infarction, pre-operative dialysis, estimated glomerular filtration rate less than 15 ml^–1^ 1.73 m^–2^, and systolic blood pressure less than 90 mmHg before anesthesia.

Patient demographics, medical history, routine pre-operative laboratory examinations and surgery-related information were collected for the study, including age, sex, body mass index, hypertension, coronary artery disease, cerebral infarction, hyperlipemia, ascites, use of renin-angiotensin-aldosterone system inhibitors, diuretic use, estimated glomerular filtration rate, hemoglobin, HbA1c, albumin, total bilirubin, noradrenaline, influid amount, blood loss, American Association of Anesthesiologists (ASA) physical condition, and post-lung infection. The estimated glomerular filtration rate was used to assess pre-operative kidney function according to a modified modification of the diet in renal disease equation, the efficiency of which has been demonstrated in the Chinese population (22). It was calculated using the Formula 186 × (preoperative serum creatinine)^–1.154^ × (age)^–0.203^ × (0.742, if female) (23). HbA1c was measured by an ARKRAY automatic glycohemoglobin analyzer (ARKRAY Factory, Shanghai China) on the ADAMS A1c HA-8180 system in the medical laboratory center in our hospital. The measurement range was 3–20% or 14–191 mmol/mol. Pre-operative HbA1c could be obtained in the study population.

The primary outcome was the occurrence of AKI. AKI was diagnosed according to the criteria and grades of the Kidney Disease: Improving Global Outcomes (KDIGO) guidelines (increase in serum creatinine of ≥ 26.5 μmol l^–1^ within 48 h or ≥ 1.5 times baseline within 7 days after surgery) (24). The most recent pre-operative creatinine value was used as the baseline.

## Statistical Analysis

Continuous variables are represented as the means (standard deviation, SD), and the Mann–Whitney U test was used for comparisons. Categorical variables were expressed as counts and percentages and were compared through χ^2^ or Fisher’s exact test as needed.

To study the relationship between pre-operative HbA1c and post-operative AKI, we treated HbA1c both as a categorical (in quintiles) and as a continuous variable and performed univariable and multivariable logistic regression analysis. We adjusted part of the above baseline variables in the multivariate model because they have been identified or assumed to be risk factors for post-operative AKI. The results were reported as ORs and 95% CIs. All variables in the final multivariable model were confirmed with variance inflation factors < 2.5 to avoid multicollinearity. The shape of the multivariable association between pre-operative HbA1c and the probability of AKI occurrence was shown by a restricted cubic spline to the continuous model.

To evaluate the additional predictive value of pre-operative HbA1c beyond traditional AKI risk factors, we used the χ^2^ likelihood ratio test to determine whether the multivariable logistic regression model containing HbA1c was better than the model without it. In addition, using the DeLong method to test the changes in the area under the receiver operating characteristic curve (AUC), the ability of the models to distinguish AKI cases from non-cases can be compared (25). The Net Reclassification Index (NRI) and the Integrated Discrimination Improvement Index (IDI) were used to evaluate the risk reclassification ability of HbA1c before the operation (26, 27). We used the continuous NRI version for preliminary analysis because the NRI indicator is a more objective way to compare different studies. Two pre-operative prediction models have been established and validated for AKI in non-cardiac surgery patients, including the weighted general surgery AKI (GS-AKI) risk index (28) and the simple post-operative AKI risk (SPARK) index (29). We added pre-operative HbA1c to the models and evaluated whether it could improve the predictive ability.

Decision curve analysis was used to evaluate the usefulness of several different models. We took this approach to compare the net benefit of the models across a range of reasonable thresholds. Based on this range, clinicians can determine which patients are at high risk of post-operative AKI and adopt intervention measures for them (30). The net benefit was considered to be the difference between the proportion of patients who had true positive predictions (i.e., predicted to be at “high risk” and developed AKI) and the proportion of patients who had false-positive predictions (i.e., predicted to be at “high risk” but did not develop AKI) weighted by the odds of the selected threshold for “high risk.” Within a given threshold range, we chose the model with the larger net benefit as the most preferred.

To maximize statistical power, the sample size was not calculated for this study, and we planned to analyze all eligible patients. In logistic regression models, we used multiple imputation with chained equations, that is, 20 datasets being imputed, averaging predictions and taking into account uncertainty owing to imputation, for handling missing data (31).

R software was used for statistical analysis. All statistical tests were two-tailed.

## Results

**FIGURE 1:**
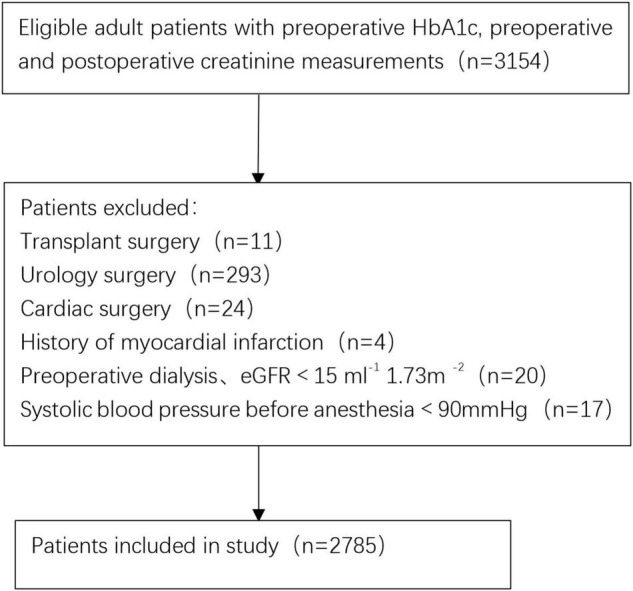
Flow chart for patients selection.

Initial screening consisted of 3,154 patients (≥ 18 years old) who underwent surgery between October 2011 and December 2020. A total of 369 patients were excluded from the final cohort based on the exclusion criteria. Figure 1 shows a flowchart of the patient selection process. Therefore, 2,785 adult patients were eligible for the study. A total of 3.3% of patients (94/2785) developed AKI within 1 week after surgery. According to the occurrence of post-operative AKI, the overall and stratified characteristics of the study are shown in Table 1. Patients who developed post-operative AKI were older and had poorer pre-operative baseline conditions.

**TABLE 1:** Baseline characteristics of the study.

Variables^a^	Overall (*n* = 2785)	No AKI (*n* = 2691)	AKI (*n* = 94)	*P*
Age, years	54.53 ± 14.17	54.32 ± 14.16	60.43 ± 13.47	< 0.001
Female	1806 (64.8)	1749 (65.0)	57 (60.6)	0.447
BMI, kg m^–2^	25.78 ± 5.39	25.81 ± 5.36	24.82 ± 5.93	0.079
**Complication**				
Hypertension	1174 (42.2)	1125 (41.8)	49 (52.1)	0.059
CAD	235 (8.4)	219 (8.1)	16 (17.0)	0.004
Cerebral infarction	214 (7.7)	199 (7.4)	15 (16.0)	0.004
Hyperlipemia	333 (12.0)	321 (11.9)	12 (12.8)	0.933
Ascites	31 (1.1)	27 (1.0)	4 (4.3)	0.014
**Medication**				
ACEI	69 (2.5)	63 (2.3)	6 (6.4)	0.032
ARB	28 (1.0)	27 (1.0)	1 (1.1)	1.000
Diuretic	55 (2.0)	51 (1.9)	4 (4.3)	0.215
**Pre-operative laboratory findings**				
eGFR, ml min^–1^ 1.73 m^–2^	98.58 ± 22.89	99.01 ± 22.26	86.2 ± 34.51	< 0.001
Hemoglobin, g L^–1^	124.33 ± 24.25	124.73 ± 24.04	112.67 ± 27.42	< 0.001
HbA1c,%	7.09 ± 1.79	7.07 ± 1.79	7.64 ± 1.75	0.002
Albumin, g L^–1^	39.74 ± 6.22	39.85 ± 6.10	36.46 ± 8.48	< 0.001
Total bilirubin (IQR), μmol^–1^	11.8 (8.8–15.9)	11.8 (8.9–15.8)	11.3 (7.5–16.95)	0.002
**Surgical characteristics**				
Noradrenaline	212 (7.6)	197 (7.3)	15 (16.0)	0.004
Influid amount (IQR), ml	1600(1100–2550)	1600(1100–2550)	2000(1312.5–2681.25)	0.115
Blood loss (IQR), ml	100 (50–300)	100 (50–300)	200 (100–500)	< 0.001
ASA physical status				< 0.001
1	93 (3.3)	93 (3.5)	0 (0.0)	
2	1432 (51.4)	1409 (52.4)	23 (24.5)	
3	1148 (41.2)	1091 (40.5)	57 (60.6)	
4	108 (3.9)	94 (3.5)	14 (14.9)	
5	4 (0.1)	4 (0.1)	0 (0.0)	
Post-lung infection	185 (6.6)	166 (6.2)	19 (20.2)	< 0.001

*Values are the mean (SD) or number (%). a: categorical variables are shown as counts (percentages), and continuous variables are shown as the means (standard deviation). AKI, acute kidney injury, BMI, body mass index, CAD, coronary artery disease, ACEI, angiotensin converting enzyme inhibitors, ARB, angiotensin receptor blocker, eGFR, estimated glomerular filtration rate, HbA1c, glycosylated hemoglobin A1c, IQR, interquartile range, ASA, American Society of Anesthesiologists.*

*^a^Categorical variables are shown as counts (percentages) and continuous variables are shown as the means (standard deviation).*

The mean and standard deviation of pre-operative HbA1c was 7.09 (1.79). All variables in the final multivariable model were confirmed to have variance inflation factors < 2. Consequently, there was no obvious collinear relationship between HbA1c and other variables.

**FIGURE 2:**
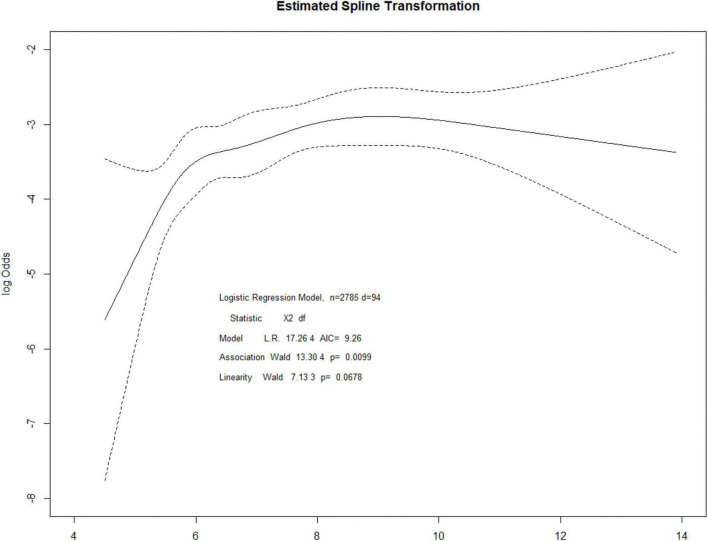
Restricted cubic spline regression for the association between HbA1c and post-operative AKI.

Patients with post-operative AKI had significantly higher pre-operative HbA1c values than those without AKI (mean 7.64 vs. 7.07, *P* = 0.002, Table 1). The risk of AKI increased across quintiles of HbA1c, with more than fourfold higher odds of AKI for the highest quintile than for the lowest quintile (Table 2). Even after adjusting some variables in logistic regression models, such as age, hemoglobin, albumin, estimated glomerular filtration rate, ASA, influid amount, blood loss, hypertension, and coronary artery disease, quintiles 3, 4, and 5 remained independently associated with post-operative AKI. Pre-operative HbA1c was also independently and significantly associated with post-operative AKI when HbA1c was included in the logistic regression model as a continuous variable (adjusted OR per percentage point increment in HbA1c 1.20, 95% CI, 1.07 to 1.33, *P* = 0.001, Table 2). A restricted cubic spline model described the relationship between pre-operative HbA1c and post-operative AKI risk (Figure 2).

**TABLE 2:** Associations of pre-operative glycosylated hemoglobin A1c with acute kidney injury after non-cardiac surgery.

	Categorized HbA1c (quintiles)						Continuous HbA1c	
	4.1–5.5	5.6–6.1	6.2–7.0	7.1–8.4	8.5–15.6	P trend	p value	
Unadjusted OR	1 (reference)	2.16 (0.82, 5.66)	3.07 (1.24, 7.59)	3.34 (1.34, 8.30)	4.46 (1.84, 10.83)	0.0005	1.16 (1.05, 1.28)	0.003
Adjusted OR^a^	1 (reference)	2.17 (0.78, 6.03)	2.88 (1.08, 7.67)	3.38 (1.26, 9.06)	5.02 (1.90, 13.24)	0.0005	1.20 (1.07,1.33)	0.001

*Values are OR (95% CI). a: adjusted for age (per year), hemoglobin, albumin, estimated glomerular filtration rate, ASA, influid amount, blood loss, hypertension, and coronary artery disease. ASA, American Society of Anesthesiologists, HbA1c, glycosylated hemoglobin A1c.*

### The Additional Value of HbA1c in Risk Prediction

The likelihood ratio test showed that adding HbA1c better improved the model for AKI prediction. The discriminant AUC of the model containing only clinical risk factors was 0.7387 (95% CI, 0.6813 to 0.7965) and 0.7543 (95% CI, 0.6956 to 0.8075) for the model containing both clinical risk factors and HbA1c. After adding HbA1c to the base model, the AUC value increased by 0.0156 (shown in Table 3), and the change in AUC was slight. Nonetheless, it resulted in a 32.2% improvement in reclassification of patients who did not develop AKI after surgery and a modest improvement in net reclassification in the overall population (continuous NRI 0.2767, 95% CI, 0.0715 to 0.4818 *P* = 0.0082). However, reclassification did not improve in patients with AKI (–0.0851 95% CI, –0.2865 to 0.1163); simultaneously, the integrated discrimination improvement was 0.0048 (95% CI, –0.0005 to 0.0101). We also explored the effect of HbA1c on other predictive models of post-operative AKI. Adding pre-operative HbA1c to the GS-AKI risk index or SPARK index could improve the model performance to a certain extent, such as a mild increase in AUC, continuous NRI and IDI index (Table 3).

**TABLE 3:** Performance metrics of acute kidney injury prediction models with and without glycosylated hemoglobin A1c.

	Basic model	Basic model + HbA1c^a^
AUC	0.7387 (0.6813–0.7965)	0.7543 (0.6956–0.8075)
ΔAUC	Reference	0.0156 *P* = 0.0545
NRI for event	Reference	–0.0851 (–0.2865–0.1163)
NRI for non-event	Reference	0.3222 (0.2864–0.3580)
NRI	Reference	0.2767 (0.0715–0.4818)
IDI	Reference	0.0048 (–0.0005–0.0101)
	SPARK	SPARK + HbA1c
AUC	0.7024 (0.6502–0.7546)	0.7158 (0.6666–0.7651)
ΔAUC	Reference	0.0134 *P* = 0.2455
NRI	Reference	0.2136 (0.0094–0.4177)
IDI	Reference	0.0015 (–0.0009–0.0039)
	GSAKI	GSAKI + HbA1c
AUC	0.6593 (0.605–0.7136)	0.6780 (0.6284–0.7316)
ΔAUC	Reference	0.0187 *P* = 0.042
NRI	Reference	0.2371 (0.0325–0.4417)
IDI	Reference	0.001 (–0.0011–0.0032)

*^a^Adjusted for age (per year), hemoglobin, albumin, estimated glomerular filtration rate, ASA, influid amount, blood loss, hypertension, and coronary artery disease. ΔAUC, change in area under the curve, ASA, American Society of Anesthesiologists, AUC, area under the curve, IDI, integral discrimination improvement, NRI, net reclassification improvement, HbA1c, glycosylated hemoglobin A1c.*

**FIGURE 3:**
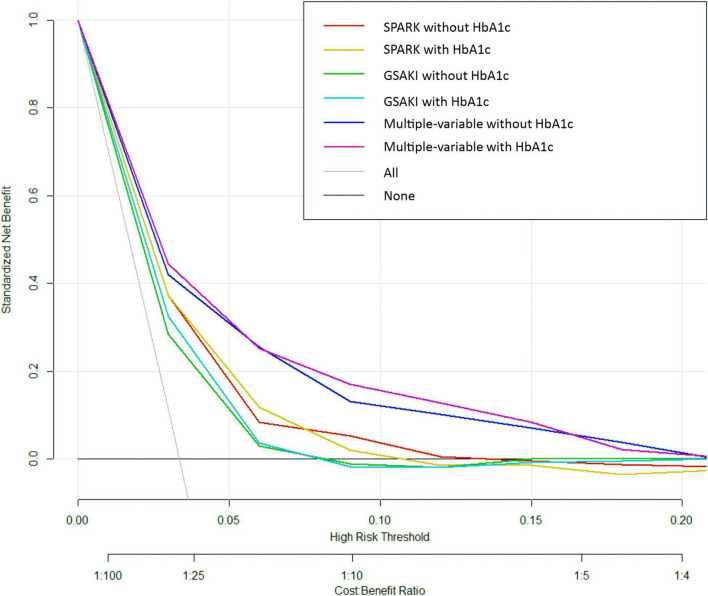
Decision curve analysis.

Finally, decision curve analysis was used to facilitate the comparison between different prediction models. As seen in Figure 3, the decision curve analysis graphically shows the clinical usefulness of each model based on a continuum of potential thresholds for post-operative AKI risk (*x* axis) and the net benefit of using the model to risk stratify patients (*y* axis) relative to assuming that no patient will have post-operative AKI. In this analysis, the multivariable model including HbA1c yielded the highest net benefit when the decision thresholds were between 2% and 16%. A threshold below 2% (i.e., one patient with AKI for every 50 patients undergoing surgery) was similar for multivariable models with and without HbA1c regarding net benefits, but the net benefits were higher than those of SPARK + HbA1c and GS-AKI + HbA1c. Beyond a decision threshold of 16% (i.e., one patient with AKI for every 6.25 patients undergoing surgery), there was little point in adding HbA1c. In the SPARK and GSAKI risk models, the models incorporating HbA1c could achieve higher net benefits within a certain threshold range.

## Discussion

In this retrospective analysis, our findings suggest that elevated HbA1c is an independent predictor of post-operative AKI in non-cardiac surgery patients. In a multivariable model constructed from traditional AKI risk factors such as demographics, laboratory tests, and surgery-related variables, adding HbA1c to build a new model was found to improve the net reclassification index and the integrated discrimination improvement to some extent. When the decision threshold range is between 2% and 16%, a new predictive model with HbA1c can acquire more net benefits. These results indicate that HbA1c plays an important predictive role in AKI risk assessment in patients who have undergone non-cardiac surgery.

Several studies have indicated that elevated HbA1c was associated with increased renal complications and was a predictor of early AKI after cardiac surgery. Pre-operative HbA1c levels (> 6.0%) were independently related to the risk of early mortality and AKI after elective cardiac surgery in a retrospective observational study (19). The findings of Halkos et al. suggested that patients with elevated HbA1c levels were closely related to adverse events after coronary artery bypass grafting, and AKI was more common in patients with higher HbA1c levels (16). Perioperative results from coronary artery bypass grafting (CABG) showed that every 1% increase in HbA1c above 5.9% was correlated with a 23.6% increased risk of renal complications (18). The type of surgery was one of the risk factors for post-operative AKI. Patients undergoing cardiac surgery were prone to hemodynamic changes and were vulnerable to post-operative AKI risk. Research showed that the occurrence of AKI was up to 15% in patients who underwent cardiopulmonary bypass (CPB) during cardiac major vascular surgery, and in general surgery patients, the incidence of AKI was 1% (28). However, the predictive power of HbA1c for AKI in patients undergoing non-cardiac surgery remains unclear. Our findings expanded previous observations by demonstrating that pre-operative HbA1c can also predict AKI after non-cardiac surgery, and the association remained evident after adjusting for traditional AKI risk factors. This study extended the findings of a recent study that elevated HbA1c was a predictor of AKI after minimally invasive nephrectomy (8). The mechanism of AKI in patients with poor glycemic control (elevated HbA1c) is unclear. It has been suggested that advanced glycation end products may be important in the development of arteriosclerosis (32, 33), there is evidence that elevated HbA1c is associated with microvascular and macrovascular events (34), and a study has shown that glycosylation changes in the mesangial matrix are most likely to contribute to post-operative AKI in patients with elevated HbA1c (35). Therefore, the prognostic value of HbA1c may be related to changes in glycosylation products and the vascular endothelium.

The new model with the addition of HbA1c improved the discrimination ability of post-operative AKI compared to traditional risk factor models. The absolute change in AUC (0.0156) for the two models may seem small, but AUC was an insensitive metric for evaluating new predictors. Even though the model had important improvements in risk forecasting, the AUC may change very little (36). Improvements in the predictive efficiency of the new model were also supported by NRI and IDI and decision curve analysis. The advantage of the new model with the addition of HbA1c shown by the NRI was primarily its ability to reclassify patients who did not develop AKI into the low-risk group. In decision curve analysis, the horizontal curve showed that no one was intervened, and the net benefit was zero. The sloping curve meant that everyone accepted the intervention, and the net benefit was a backslash with a negative slope. The multivariate model with HbA1c was far from the two extreme curves in the figure; therefore, it could provide the best net benefit to the patients. Decision curve analysis indicated the predictive value of HbA1c from the perspective of clinical applicability.

Despite some statistical significance, the new model performance showed only a modest improvement. Several other biomarkers have begun to be suggested for predicting perioperative AKI (37–39). Individually, these markers may have little effect on traditional risk factors; perhaps a combination of some markers could enable greater model improvement, and they may lead to potential interventions for AKI from different pathophysiological aspects.

This is clinically valuable when biomarkers can help identify and improve outcomes in high-risk patients (40). At present, some kidney biomarkers are being researched, including insulin-like growth factor-binding protein 7 (IGFBP7), tissue inhibitor of metalloproteinases-2 (TIMP-2), neutrophil gelatinase-associated lipocalin (NGAL), and liver-type fatty acid-binding protein (LFAP). Urine and serum NGAL concentrations were early predictive biomarkers of AKI after cardiac surgery (41). L-FABP was approved for use in predicting AKI in Japan (42). It was suggested that urinary (TIMP-2) × (IGFBP7) is a sensitive and specific biomarker to predict early AKI and renal recovery after cardiac surgery (43). Biomarkers have already been used to guide treatment for patients who are prone to AKI. Urine (TIMP-2) × (IGFBP7) > 0.3 was used to define patients at high risk of post-operative AKI and to guide intensive care in KDIGO guidelines, including volume optimization, hemodynamic stability, avoidance of nephrotoxic drugs, prevention of hyperglycemia, etc. It reduced the incidence and severity of post-operative AKI compared to standard care (44). A quality initiative program (45) showed that implementing supportive measures in biomarker-positive patients can reduce the incidence of moderate and severe AKI after cardiac surgery (46) and abdominal surgery (47). However, these markers began to increase after kidney damage occurred, allowing early detection but not prediction of AKI. Clinicians cannot use these to guide pre-operative and intraoperative management. Whether early prediction and prevention based on HbA1c can lead to greater efficacy in reducing post-operative AKI is unclear. While our decision curve analysis shows that using HbA1c increases net benefit, this analysis is preliminary and is not a substitute for a randomized trial. In addition, a formal cost–benefit analysis is required to weigh the additional cost of the study against the benefit of applying it to direct clinical management.

There were some limitations in this research. First, the study type was retrospective, with potential selection bias. Second, even if multiple variables were controlled in the analysis, confounders may still exist. We cannot rule out some other factors that were not routinely measured prior to surgery, such as new markers of kidney injury and inflammation, which may be more strongly associated with post-operative AKI than HbA1c. Third, in this study, we focused on patients at risk for AKI through pre-operative identification. Pre-operative clinical variables or biomarkers have difficulty accurately predicting multifactorial post-operative complications such as AKI. Intraoperative and post-operative variables, such as hypovolemia and the use of nephrotoxic drugs, were also major risk factors for AKI. These unpredictable factors may make any pre-operative predictive model partially imprecise if models include both pre-operative and post-operative factors, which can improve the predictive performance. However, the clinical efficiency of this kind of prediction model may also be lower.

## Conclusion

In this retrospective cohort study of 2,785 patients undergoing non-cardiac surgery, elevated pre-operative HbA1c was independently associated with post-operative AKI risk and had auxiliary predictive value. HbA1c improves the predictive power of a logistic regression model based on traditional clinical risk factors for AKI. The clinical applicability of the findings requires further studies to verify whether pre-operative assessment and treatment based on HbA1c can reduce the incidence of post-operative AKI.

## Data Availability Statement

The original contributions presented in this study are included in the article/Supplementary Material, further inquiries can be directed to the corresponding authors.

## Ethics Statement

This study was reviewed and approved by the Ethics Committee of the Third Xiangya Hospital, Central South University. Because it was a retrospective observatory study, written informed consent was not applicable.

## Author Contributions

YL and Y-ZT contributed to the conception and design of the study. KP performed data collection and statistical analysis. L-PW collated and interpreted the results and wrote the first draft of the manuscript. BL helped to revise the manuscript and edit the typographic errors. All authors approved the submitted version.

## Conflict of Interest

The authors declare that the research was conducted in the absence of any commercial or financial relationships that could be construed as a potential conflict of interest.

## Publisher’s Note

All claims expressed in this article are solely those of the authors and do not necessarily represent those of their affiliated organizations, or those of the publisher, the editors and the reviewers. Any product that may be evaluated in this article, or claim that may be made by its manufacturer, is not guaranteed or endorsed by the publisher.
