# Supplementary material for: Predictive Value of Glycosylated Hemoglobin for Post-operative Acute Kidney Injury in Non-cardiac Surgery Patients
Source: Front Med (Lausanne). 2022 Jul 11;9:886210. doi: 10.3389/fmed.2022.886210 (PMC9309303; doi:10.3389/fmed.2022.886210)
Supplement: Supplementary file 1 [file Image_1.pdf]

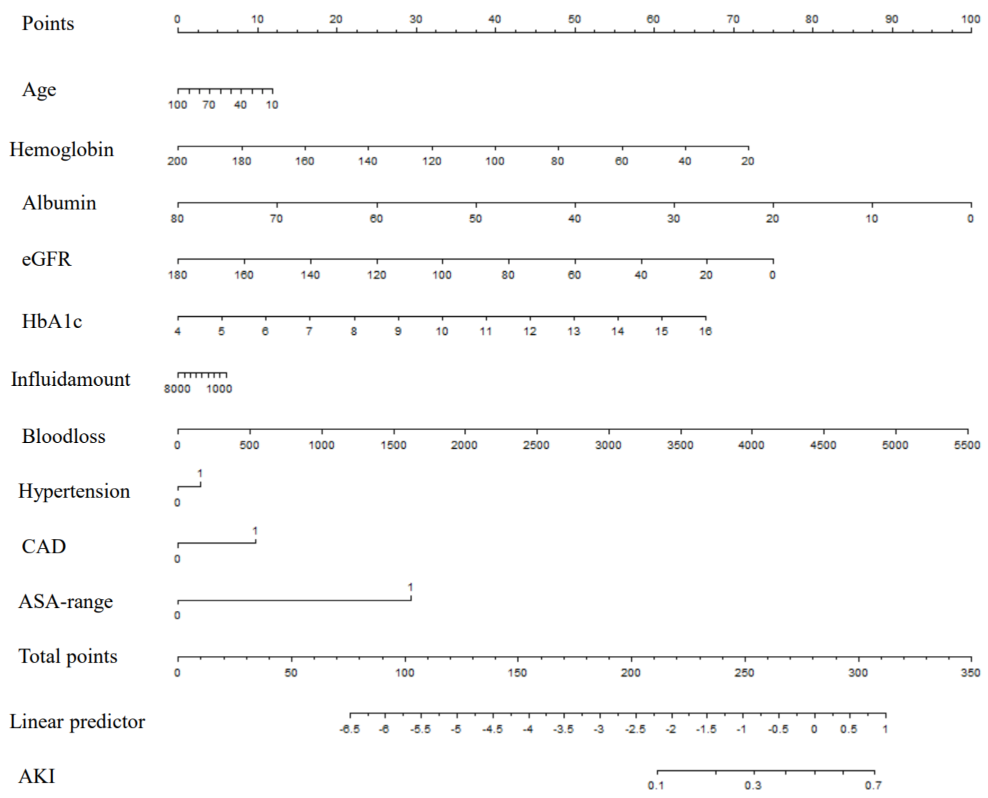

Nomogram

eGFR, estimated glomerular filtration rate, HbA1c, glycosylated hemoglobin A1c, CAD, coronary artery disease, ASA, American Society of Anesthesiologists, AKI, acute kidney injury
