# Supplementary material for: Predictive Value of Glycosylated Hemoglobin for Post-operative Acute Kidney Injury in Non-cardiac Surgery Patients
Source: Front Med (Lausanne). 2022 Jul 11;9:886210. doi: 10.3389/fmed.2022.886210 (PMC9309303; doi:10.3389/fmed.2022.886210)
Supplement: Supplementary file 2 [file Table_1.docx]

| Variables | Missing percentage | Missing number |
| --- | --- | --- |
| Age | 0.78% | 24 |
| Female | 0.21% | 6 |
| ACEI | 0.28% | 8 |
| ARB | 0.28% | 8 |
| eGFR | 5.62% | 177 |
| Hemoglobin | 2.13% | 63 |
| Albumin | 2.79% | 80 |
| Total bilirubin | 2.79% | 80 |
| Influid amount | 0.17% | 5 |
| Blood loss | 4.09% | 128 |

Missing variables table

ACEI, angiotensin converting enzyme inhibitors, ARB, angiotensin receptor blocker, eGFR, estimated glomerular filtration rate,
